# Autoantibodies in association with subchorionic haematoma in early pregnancy

**DOI:** 10.1080/07853890.2021.1936150

**Published:** 2021-06-04

**Authors:** Yang Li, Ensheng Wang, Shisi Huang, Changling Zhu, Kemei Zhang, Jiaou Zhang, Haiyan Xu, Jing Shu

**Affiliations:** Reproductive Medicine Center, Ningbo First Hospital, Ningbo, China

**Keywords:** Subchorionic haematoma, autoantibody, pregnancy outcome

## Abstract

**Objective:**

To explore the possible aetiology of subchorionic haematoma (SCH), especially its association with autoantibodies.

**Material and methods:**

Early pregnant women who were detected SCH through ultrasonography were identified as the study group and those without SCH at comparable ages who visited the clinic at the same period of time were compared as the control group. Indexes of laboratory immune tests were compared between the two groups, as well as their pregnancy outcomes.

**Results:**

A total of 97 SCH patients and 130 control cases were recruited in this study. A higher proportion of women was detected autoantibodies in the SCH group compared with control group (45.36% vs 21.54%, *p* = .000). Positive rates of ANA (24.74% vs 10.77%, *p* = .005) and laboratory antiphospholipid antibodies (ACL, anti-β2 GP1 or LA) (25.77% vs 11.54%, *p* = .005) showed significant differences between the two groups. The incidence of vaginal bleeding was significantly higher in the SCH group (43.30% vs 20.00%, *p* = .000). While the miscarriage rates were not significantly different (17.53% vs 15.38%, *p* = .666). And there were no significant differences in terms of preterm delivery rate, caesarean section rate, birth weight and pregnancy complications. Most SCHs (96.25%) were absorbed before 20th gestational week. In the SCH group, the average birth weight was significantly lower in women with autoantibodies. Clinical features and other pregnancy outcomes showed no significant differences between SCH patients with and without autoantibodies.

**Conclusions:**

The occurrence of SCH may be associated with autoantibodies. The pregnancy outcomes were comparable between women with and without SCH.

**KEY MESSAGES**
1. Subchorionic haematoma (SCH) is increasingly commonly observed in early pregnancy period, but the aetiology is uncertain and the clinical significance of SCH is controversial.
2. The occurrence of SCH may be associated with autoantibodies.
3. The pregnancy outcomes were not significantly different between women with and without SCH.

## Introduction

**Figure 1.**
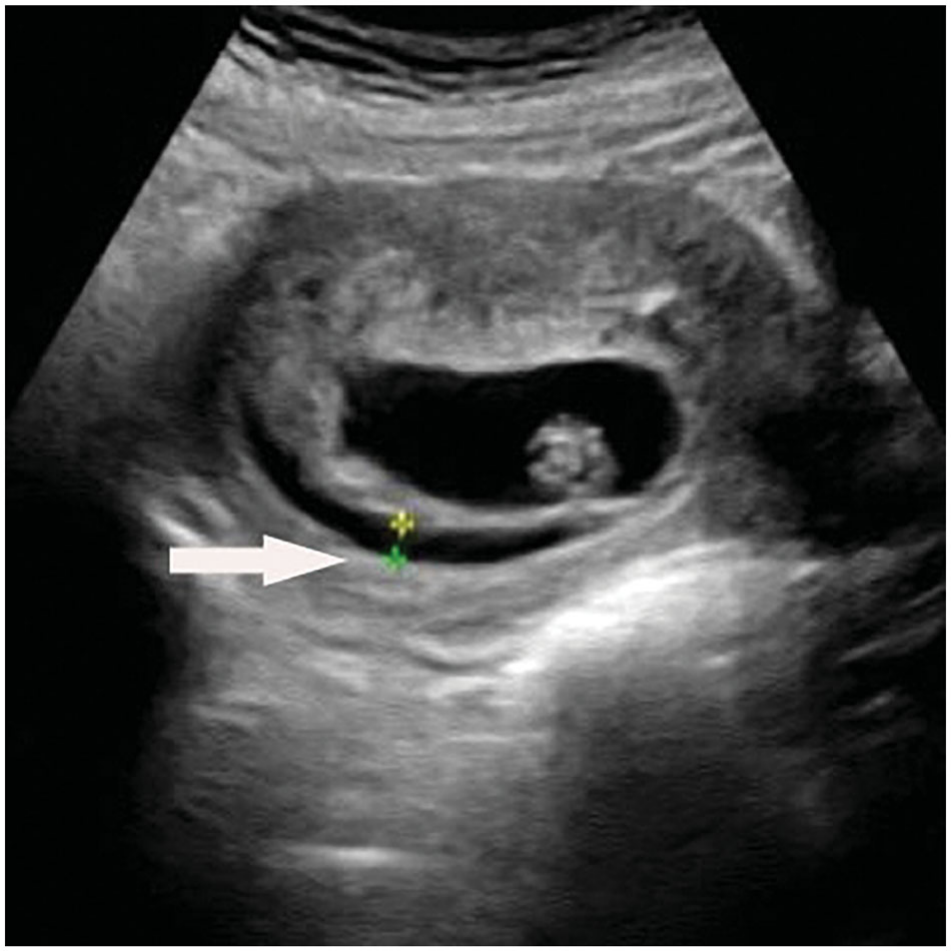
Subchorionic haematoma in the first trimester. A hypoechoic crescent-shaped area separates the uterine wall and chorion (arrow).

Tremendous improvement in medical ultrasonics lead to more precise diagnosis in obstetrics and gynaecology, with more subtle abnormalities being found. Subchorionic haematoma (SCH) is increasingly commonly observed in early pregnancy period, especially in women with vaginal bleeding. The incidence has been reported to be from 0.46% to 39.5% [1,2], depending on the populations studied and gestational age at diagnosis. The appearance of SCH on ultrasonic image is usually manifested as hypoechoic or anechoic crescent-shaped area (Figure 1). They are thought to result from partial detachment of the chorionic membrane from the uterine wall [3]. However, the exact aetiology is uncertain, and the clinical significance of SCH is controversial. On the other hand, numerous studies on reproductive failure focus on the dysregulation of maternal immune responses and thrombophilic causes [4,5]. It is possible that the occurrence of SCH is related to the imbalance of maternal-foetal immune interaction, leading to decidual immune vasculitis, intravascular microthrombosis, rupture of the decidua vessels, abnormal trophoblastic invasion, and ischaemia reperfusion followed by haemorrhage. Tuuli et al. proposed that shallow trophoblast invasion and impaired angiogenesis with resultant friable blood vessels may predispose to subchorionic haemorrhage as well as adverse outcomes [6]. The aim of the present study was to explore the possible aetiology of SCH, especially its association with autoantibodies, as well as the pregnancy outcomes of SCH patients.

## Patients and methods

### Patients

Pregnant women who visited the clinic of Reproductive Medicine Centre of Ningbo First Hospital, China from July 2018 to February 2020 were studied. They underwent ultrasound examination using a 5 MHz∼9MHz transvaginal probe (GE Voluson E8; GE Healthcare Austria GmbH & Co OG, Zipf, Austria) before 7 weeks of gestational age or a 3.5 MHz∼5MHz abdominal probe after 7 weeks. Those who were detected SCH were identified as the study group when meeting the inclusion criteria. Among those without SCH, women of comparable ages were randomly recruited as the control group when they met the inclusion criteria, excluding those who met the exclusion criteria, as well as those without complete information or who refused to undergo examinations or follow-up. The inclusion criteria were intrauterine singleton pregnancy, gestational age between 6 weeks to 16 weeks, with or without vaginal bleeding. The exclusion criteria were history of severe external forces, multiple pregnancy, uterine anomaly, foetal anomaly diagnosed through non-invasive prenatal screening, severe medical and surgical pathology. Data of general characteristics of the patients were recorded at their first visits. During the study, those who were lost to follow-up or lack relevant data were excluded. The study was approved by the ethics committee of Ningbo First Hospital. Written informed consent was obtained from all the participants.

### Study design

All the recruited patients took non-fasting venous blood tests of immune indexes in their early pregnancy periods. The test items included antinuclear antibodies (ANAs), anticardiolipin antibody (ACL), anti-β2 glycoprotein1 (anti-β2GP1) antibody, lupus anticoagulant (LA) and so on. Antinuclear antibodies were tested with an indirect fluorescent antibody method using test kits supplied by EUROIMMUN (Hangzhou) Medical Laboratory Diagnosis Co., Ltd., China. Serum fluorescence titre ≥ 1:100 was considered positive. Anticardiolipin antibodies were tested with a standardized enzyme linked immunosorbent assay (ELISA). Reagents were supplied by Shenzhen SCIARRAY Biotechnology Co., Ltd., China. ACL-IgG/IgM > 12 GPL/MPL was considered positive. Anti-β2GP1 antibodies were analysed using ELISA with test kits supplied by Guangzhou Kangrun Biological Technology Co., Ltd., China. A titre > 20 U/ml was considered positive. The definition of a moderate-to-high titre of ACL or anti-β2GP1 was 40 or more GPL or MPL units (>99th percentile). Lupus anticoagulant was tested with HemosIL dRVVT Screen and dRVVT Confirm assays from Instrumentation Laboratory Co., USA. Results of screen and confirm assays were expressed as ratios of patient to reference plasma clotting times. Confirm assay was realised when screen ratio was equal or above the cut-off value. The cut-off values for the screen and normalised ratios were 1.20.

Positive ANA may indicate some kind of autoimmune disease such as systemic lupus erythematosus (SLE) or sicca syndrome (SS) when meeting other diagnostic criteria. Otherwise, it may just indicate a state of immune imbalance, which may only make sense in the case of pregnancy. Antiphospholipid syndrome (APS) is characterised by thrombosis or/and pregnancy complications (recurrent early pregnancy loss, late pregnancy loss, or preterm labour due to severe obstetrical complications such as eclampsia, pre-eclampsia, or severe placenta dysfunction) in patients with persistent antiphospholipid antibodies (LA, ACL, or anti-β2GP1). Patients who are detected positive antiphospholipid antibodies can be diagnosed with APS when meeting both clinical and laboratory diagnostic criteria according to the revised Sapporo criteria [7]. Those with positive laboratory tests but without related symptomsmay also be considered at higher risks of thrombosis or pregnancy loss.

SCH on ultrasonic image appeared as hypoechoic or anechoic crescent-shaped or irregular area. Autoantibodies were compared between women with and without SCH. We checked the foetus and SCHs every 2–4 weeks. Patients’ clinical information and features of SCH were recorded in detail, including age, pregnancy history, mode of conception (natural or through assisted reproductive technology), size of SCH and the absorption time. Size of SCH was graded as a fraction of the gestational sac size according to the method reported by Nagy et al. [8] classifying the subchorionic haematoma as small (<20% of the gestational sac), medium (20–50% of the gestational sac), or large (>50% of the gestational sac).

All of these women were prescribed oral progesterone with or without progesterone injection according to their clinical manifestations such as vaginal bleeding and serum progesterone levels. Those who were diagnosed with APS or thrombophilia were treated with low-dose aspirin or/and low-molecular-weight heparin (LMWH) as well. Immunomudulator (hydroxychloroquine) or/and low dose anti-immune agent (prednisone) were given to patients with autoimmune disease such as SLE and SS diagnosed by rheumatologists. When patients presented large SCH or heavy vaginal bleeding, low-dose aspirin was stopped and the dose of LMWH was adjusted. We followed up the patients until delivery.

Miscarriage was diagnosed when any of the following conditions was met: persistent vaginal bleeding or abdominal pain, followed by expulsion of the embryo; failed to detect embryo’s cardiac beat for two or more ultrasonic tests after 7 weeks of gestational age; normal cardiac beat disappeared. Live birth rate, birth weight, preterm delivery rate, caesarean section rate and obstetric complications such as gestational hypertension or placental abruption were compared between the groups. For those who gave birth in our hospital, we completed the follow-up work by consulting their medical records. We made telephone follow-ups to get information of SCH’s progress and their pregnancy outcomes if they took antenatal examinations and gave births in other hospitals.

### Statistical analysis

Statistical analysis of the clinical data was performed by SPSS Statistics version 19.0 (SPSS Inc., Chicago, IL, USA). Continuous data were presented as mean ± standard deviation (SD) and compared with Student’s t-test. Categorical data were presented as proportion (percentage) and compared with Chi-square test or Fisher’s exact test where appropriate. *p*-Values of <.05 were considered statistically significant.

## Results

A total of 102 women were detected SCH. Three were lost to follow-up and two women had twins so excluded from the research. So the study group included 97 cases. Eliminating those who were lost to follow-up or without sufficient information, the control group included 130 women without SCH. There were no statistically significant differences between the two groups in terms of age, previous pregnancy loss history and childbirth history (Table 1). There was a larger percentage of in-vitro fertilization (IVF) pregnancy in the SCH group than in the control group (*p* = .025).

**Table 1.** Characteristics of pregnant women with and without SCH.

	With SCH *n* = 97	Without SCH *n* = 130	*p*-Value
Age (years)	29.71 ± 3.61	29.96 ± 3.14	.578
Patients with pregnancy loss history [*n* (%)]	46 (47.42)	51 (39.23)	.217
—One pregnancy loss [*n* (%)]	30 (30.93)	33 (25.38)	.356
—Recurrent pregnancy loss [*n* (%)]	16 (16.49)	18 (13.85)	.580
Multiparous [*n* (%)]	11 (11.34)	12 (9.23)	.602
Mode of conception			
—Natural [*n* (%)]	87 (89.69)	126 (96.92)	.025
—IVF [*n* (%)]	10 (10.31)	4 (3.08)	
Positive autoantibodies [*n* (%)]	44 (45.36)	28 (21.54)	.000
—ANA [*n* (%)]	24 (24.74)	14 (10.77)	.005
Titre ≥1:320	10 (10.31)	4 (3.08)	.025^a^
Titre =1:100	14 (14.43)	10 (7.69)	
—ACL [*n* (%)]	5 (5.15)	4 (3.08)	.502
Moderate-to-high titre	1 (1.03)	0	.427^a^
Low titre	4 (4.12)	4 (3.08)	
—Anti-β2GP1 [*n* (%)]	12 (12.37)	4 (3.08)	.007
Moderate-to-high titre	5 (5.15)	1 (0.77)	.086^a^
Low titre	7 (7.22)	3 (2.31)	
—LA [*n* (%)]	12 (12.37)	8 (6.15)	.102
—ACL or anti-β2GP1 or LA [*n* (%)]	25 (25.77)	15 (11.54)	.005
Clinically diagnosed autoimmune disease [*n* (%)]	9 (9.28)	3 (2.31)	.020
—SLE	1 (1.03)	0	.427
—SS	2 (2.06)	1 (0.77)	.577
—UCTD	3 (3.09)	1 (0.77)	.316
—APS	3 (3.09)	1 (0.77)	.316
Vaginal bleeding [*n* (%)]	42 (43.30)	26 (20.00)	.000

Data were presented as mean ± standard deviation (SD) or number (%).

SCH: subchorionic haematoma; IVF: in-vitro fertilisation; ANA: antinuclear antibody; ACL: anticardiolipin antibody; anti-β2GP1: anti-β2 glycoprotein1; LA: lupus anticoagulant. SLE: systemic lupus erythematosus; SS: sicca syndrome, UCTD: undifferentiated connective tissue disease; APS: antiphospholipid syndrome.

^a^Chi-square test or Fisher’s exact test were performed comparing high titre of the autoantibodies between the two groups.

The results of immune tests in the first trimester between women with and without SCH were compared (Table 1). More cases in the SCH group were detected positive autoantibodies in laboratory immune tests (45.36% vs 21.54%, *p* = .000). Positive rates of ANA (24.74% vs 10.77%, *p* = .005) and laboratory antiphospholipid antibodies (ACL, anti-β2 GP1 or LA) (25.77% vs 11.54%, *p* = .005) showed significant differences between the two groups. Different titres of ANA (1:100 or ≥1:320), ACL (low titre or moderate-to-high titre) and anti-β2GP1 (low titre or moderate-to-high titre) were analysed. Positive rate of ANA with a high titre (≥1:320) was significant higher in the SCH group, while presence of a moderate-to-high titre ACL or anti-β2GP1 did not show significant differences between the groups. Combining clinical symptoms with these laboratory tests, some patients were diagnosed with some kind of autoimmune disease such as SLE, SS, undifferentiated connective tissue disease (UCTD), APS. The total case numbers of these disorders were significant different between the two groups, but failed to show differences separately. The incidence of vaginal bleeding was significantly higher in the SCH group (43.30% vs 20.00%, *p* = .000). However, the miscarriage rates were not significantly different between them (17.53% vs 15.38%, *p* = .666) (Table 2). Final pregnancy outcomes were compared, including preterm delivery rate, caesarean section rate, birth weight, percentage of small for gestational age (SGA) newborn and pregnancy complications (Table 2). There were no significant differences in terms of these outcomes between the two groups.

**Table 2.** Pregnancy outcomes of women with and without SCH.

	With SCH *n* = 97	Without SCH *n* = 130	*p*-Value
Miscarriage [*n* (%)]	17/97 (17.53)	20/130 (15.38)	.666
Preterm delivery [*n* (%)]	10/80 (12.50)	8/110 (7.27)	.224
C-section [*n* (%)]	40/80 (50.00)	59/110 (53.64)	.620
Birth weight (g)	3138.63 ± 505.59	3260.27 ± 486.00	.096
SGA [*n* (%)]	3/80 (3.75)	3/110 (2.73)	.698
Obstetric complications [*n* (%)]			
—Diabetes	9/80 (11.25)	10/110 (9.09)	.624
—Hypertension	3/80 (3.75)	1/110 (0.91)	.312
—Abruption	1/80 (1.25)	2/110 (1.82)	1.000
—Premature rupture of membrane	6/80 (7.50)	7/110 (6.36)	.759
—Oligohydramnios	4/80 (5.00)	5/110 (4.55)	1.000
—thrombocytopenia	2/80 (2.50)	1/110 (0.91)	.574
—hyperbilirubinemia	1/80 (1.25)	1/110 (0.91)	1.000

Data were presented as number (%) or mean ± standard deviation (SD).

SCH: subchorionic haematoma; C-section: caesarean section; SGA: small for gestational age.

Features of SCH were studied in detail. The gestational age when first observed SCH was 7.82 ± 2.12 (5–14) weeks. 56.70% of SCH were calculated as small, 20.62% were medium and 22.68% were large when first observed. Evaluating the largest size during pregnancy, the three grades included 45.36%, 21.65% and 32.99%, respectively. Most SCHs (96.25%) were absorbed before 20th gestational week. One case persistently existed until delivery.

Table 3 showed the comparison of SCH patients with and without autoantibodies. There were no significant differences in terms of gestational age when SCH was first observed, SCH size, gestational age when SCH was absorbed, duration of SCH and the incidence of vaginal bleeding. Miscarriage rate, preterm delivery rate and percentage of SGA between the two groups also showed no significant differences, although the average birth weight was significantly lower in women with autoantibodies (*p* = .018).

**Table 3.** Comparison between SCH patients with and without autoantibodies.

	Autoantibody positive *n* = 44	Autoantibody negative *n* = 53	*p*-Value
Age (years)	29.30 ± 3.41	30.06 ± 3.76	.304
Gestational age when SCH first appeared (weeks)	8.06 ± 2.30	7.62 ± 1.96	.318
Grading of SCH according to its largest size [*n* (%)]^a^			
Small	15 (34.09)	29 (54.72)	.122
Medium	11 (25.00)	10 (18.87)	
Large	18 (40.91)	14 (26.42)	
Gestational age when SCH was absorbed (weeks)^b^	14.60 ± 4.28 (*n* = 37)	13.74 ± 3.99 (*n* = 42)	.361
Duration of SCH (weeks)^b^	6.47 ± 4.26 (*n* = 37)	5.93 ± 3.65 (*n* = 42)	.543
Vaginal bleeding [*n* (%)]	20 (45.45)	22 (41.51)	.696
Miscarriage [*n* (%)]	6/44 (13.64)	11/53 (20.75)	.359
Preterm delivery [*n* (%)]	6/38 (15.79)	4/42 (9.52)	.505
C-section [*n* (%)]	16/38 (42.11)	24/42 (57.14)	.179
Birth weight (g)	2998.95 ± 461.30 (*n* = 38)	3265.00 ± 515.81 (*n* = 42)	.018
SGA [*n* (%)]	3/38 (7.89)	0	.103

Data were presented as mean ± standard deviation (SD) or number (%).

^a^SCH grading: small: <20% of the gestational sac, medium: 20–50% of the gestational sac, large: > 50% of the gestational sac.

^b^Gestational age when SCH was absorbed and duration of SCH were compared in live birth women excluding one case with SCH existing until delivery.

## Discussion

Ultrasound examination is necessary in early pregnancy diagnosis, with the purpose to confirm location and number of gestational sac, foetal vitality, as well as abnormal conditions during pregnancy. Subchorionic haemorrhage or haematoma is one of the abnormal ultrasonic manifestations. In a large retrospective study, 1081 out of 63,966 (1.7%) women before 22 weeks gestation were reported to have SCH [9]. Although several studies focussed on the risk factor and pathogenic mechanism, they are still puzzling. Study by Biesiada et al. presented higher portion of multiparas and more often of a pregnancy loss history in SCH women [10]. Data in the present study didn’t identify with either of those results. Asato proposed that subchorionic haematoma occurred more frequently in IVF pregnancy [11], indicating assisted reproductive technology may be one of SCH’s risk factors. In our study, the SCH group had a larger percentage of IVF pregnancy cases than the control group. Since the sample size of IVF was small, we considered the evidence was insufficient. More data are needed to explore the impact of IVF on the incidence of SCH in the future.

Pregnancy is a process in which a mother tolerates a semi-allogeneic organism. Successful pregnancy depends on coordinated interaction between the endocrine system and immune system. It has been postulated that thrombotic and inflammatory processes play an important role in the pathogenesis of pregnancy loss [5]. Immune cell populations and cytokine environment have been illustrated to be key players in the maternal response to the embryo, including balance between T-helper 1 and T-helper 2 cells, and NK cell population [12–14]. Women with autoimmune diseases or APS suffer pregnancy loss more often. It has been proposed a long time ago that subchorionic haematoma might be associated with autoantibodies, although with a small number of cases [15]. The author supposed these antibodies might increase the tendency of platelets to aggregate, which lead to thrombosis and/or vasculitis and thereby to an increased likelihood of a subchorionic haematoma [15]. In another study, Alijotas et al. concluded that in women with poor obstetric histories, autoantibodies, especially antiphospholid antibodies, might play a role in the intrauterine hematomas (IUH) development [16]. They treated those IUH patients with aspirin plus enoxaparin, obtaining good responses in an IUH resolution and pregnancy outcomes [16]. While Ashley et al. reported that taking low-dose aspirin might be associated with an increased risk of developing an SCH during the first trimester [17]. Occurrence of large SCH in women with coagulation factor deficiency has been reported in an early study [18]. Rhodes et al. proposed that SCH was presumably caused by low pressure bleeds resulting from a tear in the marginal veins of the placenta [19]. In our study, higher portion of patients in the SCH group was detected positive autoantibodies, including ANA, ACL, anti-β2GP1 and LA, indicating that the presence of autoantibodies and prethrombotic state may be an etiologic factor of SCH, in line with those previous hypothesis and studies. With the existence of autoimmune antibodies or antiphospholipid antibodies, stability of maternal-foetal interface was disrupted. They may lead to microthrombosis, microvascular rupture, thus more likely to cause SCH. A high titre of ANA, which often indicates more serious immune disorder,seemed to be more likely to increase the risk of SCH in our study. While a moderate-to-high titre of ACL or anti-β2GP1 did not show significant differences between the two groups in the results. It is likely that even low titre of antiphospholipid antibodies can increase the risk of SCH, although the results may due to the limited case number.

The impact of SCH on pregnancy outcome is controversial. Miscarriage rates were not significantly different between women with and without SCH in our study, differing from some other studies [6,8,20], although the incidence of vaginal bleeding was significantly higher in the SCH group. According to the results of a meta-analysis, the presence of SCH increased the risk of early or late pregnancy loss by 2-fold [6]. However, in a prospective cohort study, 342 pregnant women with vaginal bleeding between 9 and 20 weeks of gestational age were followed up, no association between the presence of SCH and miscarriage or preterm delivery risks was found [21]. Another retrospective case-control study by Johns et al. reported that first-trimester vaginal bleeding was associated with adverse pregnancy outcomes, but the presence of SCH had no effect on the prognosis [22]. In a word, the clinical significance of SCH is still uncertain. Our patients were prescribed progesterone, as well as anticoagulant or anti-immune agents if they were diagnosed with thrombophilia or autoimmune disorder. These treatments might improve the prognosis of SCH. On the other hand, as the existence of autoantibodies seems to be a possible cause of SCH, these drugs may be potential treatments for SCH and give the gynaecologists more choices.

Data of SCH group showed that features of SCH and the pregnancy outcomes such as miscarriage rate, preterm delivery rate and percentage of SGA did not differ between women with and without autoantibodies, except for lower birth weight in the subgroup of positive autoantibodies which may due to the relatively high preterm delivery rate or the relatively small gestational age. The limited sample size and the therapeutic effect may be factors influencing the outcomes. Anticoagulant and low-dose anti-immune agent have been proven to improve the outcomes of antiphospholipid syndrome or autoimmune disease [23–26]. They are also popular in the treatment of unexplained recurrent pregnancy loss. Ascertaining the clinical significance of SCH and the effectiveness of the therapeutic regimen needs more data and better-designed prospective studies. In addition, fundamental studies on the mechanisms of SCH, especially the correlation between SCH and autoantibodies will strengthen our understanding of the disease, which need researchers’ more efforts.

In conclusion, the occurrence of SCH may be associated with autoantibodies. In this study, the pregnancy outcomes were comparable between women with and without SCH.

## Author contributions

Y. Li and J. Shu designed the study. All the authors made the clinical diagnosis and management. Y. Li, ES Wang and SS Huang performed data collection. Y. Li analysed the data and drafted the paper. Y. Li and J. Shu revised the manuscriptcritically. All the authors have read and approved the final version of the manuscript and agreed to be accountable for all aspects of the work.

## Data Availability

Data will be shared on request to the corresponding author with permission of Ningbo First Hospital.
